# Psychological disturbances encountered by the healthcare professionals, military professionals and general public in Sri Lanka during COVID-19 pandemic: a cross-sectional study

**DOI:** 10.1186/s12888-023-04918-2

**Published:** 2023-06-21

**Authors:** Thamudi Darshi Sundarapperuma, Madushika Wishvanie Kodagoda Gamage, Nirmala Rathnayake, Eranthi Bimalee Weeratunga, Hemamali Madhushanthi Hirimbura Jagodage

**Affiliations:** Department of Nursing, Faculty of Allied Health Sciences, University of Ruhuna, Ruhuna, Sri Lanka

**Keywords:** Anxiety, Depression, Healthcare Professionals, Military professionals, General public, COVID- 19, Sri Lanka

## Abstract

**Background:**

The COVID-19 pandemic is a serious global health concern, posing a greater risk of psychological vulnerabilities for frontline healthcare workers (HCWs), military professionals and the general public around the globe. These psychological issues appear to be long lasting and heighten the risk of mental health disorders. Therefore, this study aimed to identify psychological problems encountered by HCWs, military professionals, and the general public in Sri Lanka during the COVID-19 pandemic.

**Methods:**

A descriptive cross-sectional study was undertaken with 367 participants, including frontline HCWs (n = 128), military professionals (n = 102), and the general public (n = 137). Depression and anxiety were assessed using the Peradeniya Depression Scale (PDS) and the Generalized Anxiety Disorder scale (GAD-7), respectively.

**Results:**

Mean (± SD) age of the participants was 35.0 (± 10.6) years. A reasonable proportion of participants experienced depressive symptoms (39.25%, n = 144) and severe anxiety (12.8%, n = 47). Military professionals showed depressive symptoms (73.50%, n = 75) and severe anxiety (32.4%, n = 33) predominantly. Multivariate binary logistic regression revealed that only the level of education and professional engagement affected depressive symptoms and severe anxiety (p < 0.01). Having a high level of education was a protective factor for depressive symptoms (Adjusted OR = 0.34) compared to lower-level education, while being a HCW (Adjusted OR = 4.40) and military professional (Adjusted OR = 5.43) were identified as risk factors for depressive symptoms compared to the general public. Similarly, having a high level of education was a protective factor for severe anxiety (Adjusted OR = 0.29) compared to lower-level education, while being a HCW (Adjusted OR = 3.90) and military professional (Adjusted OR = 4.52) were identified as risk factors for severe anxiety compared to the general public.

**Conclusion:**

The current study revealed a greater level of anxiety and depressive symptoms among frontline HCWs and military professionals in Sri Lanka during the pandemic of COVID-19 compared to the general public. Therefore, providing psychological first aid for them to better deal with mental problems and an emergency preparedness plan to deal with sudden outbreaks of infectious situations are important.

**Supplementary Information:**

The online version contains supplementary material available at 10.1186/s12888-023-04918-2.

## Introduction

The novel coronavirus disease (COVID-19) has considerably affected the healthcare system, economies, and societies, directly affecting the general population’s ordinary life worldwide. The pandemic caused a tremendous impact on societal functioning at many levels, affecting their jobs, businesses, income, education, family bonds and socialization from neonates to older people [1]. The general public experienced a wide range of difficulties and burdens during the pandemic; it disrupted the daily routines and duties of many frontline workforces, including healthcare workers (HCWs) and military professionals. All these factors cumulatively contributed to reducing the quality of life of people, enhancing anxiety and other psychological impairments.

The Sri Lankan government, with the support of the Ministry of Health and Tri forces, initiated a new task force to take measures to battle the COVID-19 during the pandemic [2]. HCWs and military professionals were in key positions in this battle contributing immensely.

Frontline health staff, especially those directly working with confirmed cases, are more likely to experience stigmatization and fear of spreading the disease. They are more vulnerable to adverse psychological outcomes due to long working hours, risk of infections, shortage of protective equipment, loneliness, physical fatigue, and separation from families [3–5]. Moreover, the changes in usual practice due to COVID-19 (i.e., requests to be in workplaces other than their usual) may heighten psychological problems. Increased anxiety and stress in HCWs have been associated with a range of negative outcomes, such as impaired quality of life, poor job satisfaction, and increased absenteeism [6]. Evidence suggest being a female nurse [7], having previous psychological disturbances [8, 9], being younger or less experienced, being quarantined, lack of practical and social support and experiencing stigma, frequent contact with infected cases and poor perceived support as risk factors [10] for psychological distress among HCWs.

The Sri Lankan military was at the forefront of the COVID-19 prevention operations, which was a significant movement seen in Sri Lanka [2]. In the early phase of COVID-19, defense professionals were not even allowed to take leave. They also experienced long term fatigue, massive workload, and infection threat. Therefore, they missed their family drastically for months with pandemic-related stressors, while their family members were impacted with high risk for mental health issues [11]. Furthermore, this task which is beyond their designated duty might have increased their burnout and all have stemmed in high psychological pressure among military workers.

Ensuring the psychological well-being of frontline HCWs and military professionals is imperative to deliver high quality healthcare and security to the country. Further, the optimum psychological well-being of the general public is also important to maintain the societal values and indicators of good health and social standards. Therefore, it is imperative to identify specific vulnerable groups among a larger population and take supportive measures to mitigate negative effects of dealing with COVID-19 crisis.

There is a paucity of data about the psychological impact of COVID-19 among Sri Lankans, including different people such as HCWs, military professionals, etc. during the COVID-19 pandemic and only a few studies identified the psychological experience in different categories of people at a time [10, 12, 13]. Studies of similar nature have been mostly carried out in Western countries and high-income countries in Asia [3–5, 8]. Those findings cannot be extrapolated in the Asian context owing to differences in the healthcare system and management. In the Asian healthcare system, it has massive social, cultural, economic, geographical, and political diversity. Further, it has been shaped by its history and religious values across and within the countries. These factors mainly affect the psychological impairments of people live in the region. Therefore, the findings of this study could be able to extrapolate to the countries which have similar socio-cultural, economical, and political systems. To meet this need, the present study aimed at identifying the psychological disturbances encountered by the Sri Lankans during the COVID-19 pandemic focusing HCWs, military professionals and the general public.

## Materials and methods

### Study design and setting

A descriptive cross-sectional survey was conducted in Sri Lanka during the COVID-19 pandemic (between December 2021 and July 2022).

### Study participants and selection

Data were collected from 367 study participants, including HCWs (n = 128) in the clinical settings (i.e., medical officers, nurses, medical laboratory technicians and public health inspectors), frontline military professionals (n = 102) (i.e., Sri Lanka forces), and the public (n = 137). HCWs included those who involved in patient care of people with COVID-19, while frontline military professionals included those who worked directly with people in high-risk zones and quarantine and immediate care centers. The general public included family members and close contacts of confirmed and suspected patients, high-risk groups, and people living in high-risk areas in Sri Lanka. When selecting the general population, HCWs and military professionals or their close relatives were not considered in this category with the aim of minimizing effects due to professional engagement. Further, we excluded people with diagnosed physical or psychological disabilities and children less than 18 years old.

### Data collection instruments and variables

Data were collected using a self-administered questionnaire prepared as either a Google form or printed form. The printed form was distributed in person to those without internet access and to those who were unable to manage a smart device to answer the form. The questionnaire included baseline data such as age, gender, educational level, family income, number of dependents, number of children, ethnicity, and area of residence. Further, it included Peradeniya Depression Scale (PDS) [14] and Generalized Anxiety Disorder 7 scale (GAD-7) [15] to assess depressive symptoms and anxiety.

PDS evaluates the level of depression experienced by the stakeholders and is a locally developed and validated tool [14]. The PDS has shown 88.5% of sensitivity and 85.0% of specificity in detecting depressive symptoms [14]. It consists of 25 statements on somatic symptoms, biological symptoms of depression, statements related to mood, depressive cognitions, depressive behaviors, and cultural idioms of distress. Each statement requires either a yes or no response.

GAD-7 is a 7-item questionnaire [15] that assesses anxiety disorders. Content and face validity of GAD-7 was assessed before inclusion in the study, following the standard process [16] of cross-cultural adaptation; forward translation, backward translation, review by psychologist and psychiatrist, followed by a pre-test with ten general public.

### Operational definitions

General public – The general public included family members and close contacts of confirmed and suspected patients, high-risk groups, and people living in high-risk areas in Sri Lanka but not those who were working or who have close relations in healthcare or military services.

HCWs – Medical officers, nurses, medical laboratory technicians, and public health inspectors those who were involved in patient care of people with COVID-19.

Military professionals – Members of Tri forces (Army, Navy, Air Force) who worked directly with people in high-risk zones and in quarantine and intermediate care centers.

Depression – Persistent sad, anxious, or “empty” feelings; Suicidal thoughts or suicide attempt.

Anxiety – Feeling of unease, such as worry or fear, that can be mild or severe.

Sociodemographic factors - Factors such as age, gender, educational level, family income, number of dependents, number of children, ethnicity, and area of residence.

### Data analysis

PDS and GAD-7 scales were analyzed following the guidelines provided by the respective tool developers [14, 15]. A PDS score < 10 was considered as having no depressive symptoms, and ≥ 10 was considered as having depressive symptoms [14]. In the GAD-7, scores of 0–9 as no or mild anxiety, 10–14 as moderate anxiety, and 15–21 as severe anxiety were considered [15].

Descriptive statistics were used to describe the level of psychosocial disturbances while Chi-square test was used to identify associations between categorical variables (dependent variables). The sociodemographic variables were considered as the dependent variables selected based upon the previous literature [7–9]. The variables that were identified as significant with the chi square test were further tested for significance with univariate logistic regression. Further, Binary logistic regression (forward-conditional) was used to determine the exact factors associated with the presence of depressive symptoms and severe anxiety while removing the weak associations and retaining only the strong associations. The risk was determined by calculating the Adjusted Odds Ratio (OR) with 95% confidence interval (CI). In logistic regression analysis, the depressive symptoms and anxiety were further divided into two groups as no depressive symptom vs. have depressive symptoms (score 10 as the cutoff) and no or mild to moderate anxiety vs. severe anxiety (score 10 as the cutoff). When considering the sociodemographic characteristics, further categorization was done to identify the reference group *Reference category (age: <35 years* vs. ≥ 35 years; gender: male* vs. females; number of dependent: ≤ 2 members* vs. ≥ 2 members; level of education: Up to secondary education (primary and secondary education) * vs. above secondary education (beyond secondary education); family monthly income: <50,000 Sri Lankan Rupees (LKR)* vs. ≥ 50,000 LKR; and professional engagement: general public* vs. professionals; HCWs and military professionals) [*Reference category used in logistic regression].

Statistical significance was kept at p < 0.01. The data were analyzed using SPSS 21.0 version.

### Ethical considerations

Ethical clearance was obtained from the Ethics Review Committee, Faculty of Allied Health Sciences, University of Ruhuna, Sri Lanka and written informed consent was obtained before administering the questionnaire in both forms, including the Google form and printed forms.

## Results

### Sociodemographic characteristics

The mean (± SD) age of the participants were 35.0 (± 10.6) years. The majority were < 35 years of age group (60.8%, n = 223), females (51.8%, n = 190), educated above secondary education 52.3%, n = 192), and had a monthly income ≥ 50000.00 LKR (60.5%, n = 222) (Table 1).

**Table 1:** Socio-demographic characteristics of the participants (n = 367)

Variable	Category	Frequency (%)
Age	< 35 years	223 (60.8%)
	≥ 35 years	144 (39.2%)
Gender	Male	177 (48.2%)
	Female	190 (51.8%)
Number of dependents	< 2 members	178 (48.5%)
	≥ 2 members	189 (51.5%)
Level of education	Up to secondary education (primary and secondary education)	175 (47.7%)
	Above secondary education (beyond secondary education)	192 (52.3%)
*Family monthly income	< 50,000 LKR	145 (39.5%)
	≥ 50, 000 LKR	222 (60.5%)

*230 LKR = 1 USD during the period of data collection.

LKR = Sri Lankan Rupees

### Experienced Psychological disturbances

#### Depressive symptoms

Altogether, 39.2% (n = 144) of participants reported depressive symptoms. Depressive symptoms were predominantly experienced by military professionals (73.5%, n = 75), followed by 30.5% (n = 39) of HCWs and 21.9% (n = 30) of general public (Table 2).

#### Anxiety

Anxiety was observed among 55.3% (n = 203) of the study population, while severe anxiety was presented among 12.8% (n = 47) of them. Severe anxiety was mainly reported among the military professionals (32.4%, n = 33); however, 6.3% (n = 8) of HCWs and 4.4% (n = 6) of general public had severe anxiety (Table 2).

**Table 2:** Depressive symptoms and anxiety levels among the participants (n = 367)

Variable	Category	Depressive symptoms		Anxiety		
		Have Depressive symptoms n (%)	No Depressive symptoms n (%)	Severe Anxiety n (%)	Moderate Anxiety n (%)	Mild/No Anxiety n (%)
Overall sample		144 (39.2%)	223 (60.8%)	47 (12.8%)	47 (12.8%)	273 (74.4%)
Professional engagement	HCWs (n = 128)	39 (30.5%)	89 (69.5%)	8 (6.3%)	14 (10.9%)	106 (82.8%)
	Military professionals (n = 102)	75 (73.5%)	27 (26.5%)	33 (32.4%)	16 (15.7%)	53 (52%)
	General public (n = 137)	30 (21.9%)	107 (78.1)	6 (4.4%)	17 (12.4%)	114 (83.2%)

HCWs – Healthcare workers

#### Factors associated with depressive symptoms and anxiety

Chi-square test revealed that depressive symptoms were significantly associated with gender (p = 0.01), level of education (p < 0.001), and professional engagement (p < 0.001). Depressive symptoms were higher among males, who were educated below secondary level, and among military professionals (Table 3).

**Table 3:** Association between depressive symptoms and sociodemographic characteristics (n = 367)

Variable	Category	Depressive symptoms Frequency (%)	p value**
Age	*<35 years	88 (61.1%)	0.5
	≥ 35 years	56 (38.9%)	
Gender	*Male	81 (56.3%)	0.01
	Female	63 (43.8%)	
No of dependent	*< 2 members	71 (49.3%)	0.02
	≥ 2 members	73 (50.7%)	
Level of education	*Upto secondary education (primary and secondary education)	58 (40.3%)	< 0.001
	Above secondary education (beyond secondary education)	86 (59.7%)	
Family monthly income	*< 50,000 LKR	70 (48.3%)	0.11
	≥ 50, 000 LKR	74 (33.3%)	
Professional engagement	*General public	30 (20.8%)	< 0.001
	HCWs	39 (27.1%)	
	Military personnel	75 (52.1%)	

*Reference category (age: <35 years* vs. ≥ 35 years; gender: male* vs. females; number of dependent: ≤ 2 members* vs. ≥ 2 members; level of education: Upto secondary education (primary and secondary education) * vs. above secondary education (beyond secondary education); family monthly income: <50,000 LKR* vs. ≥ 50,000 LKR; and professional engagement: general public* vs. professionals; HCWs and military professionals)

**p values derived from Chi Square test of independence

HCWs – Healthcare workers, LKR- Sri Lankan Rupees

Same analysis revealed that severe anxiety was significantly associated with gender (p = 0.002), level of education (p < 0.001), and professional engagement (p < 0.001). Severe anxiety was higher among males, who were educated below secondary level, and among military professionals (Table 4).

**Table 4:** Association between anxiety and sociodemographic characteristics (n = 367)

Variable	Category	Severe anxiety n (%)	p value**
Age	*<35 years	32(68.1%)	0.34
	≥ 35 years	15(31.9%)	
Gender	*Male	31(66.0%)	0.002
	Female	16(34.0%)	
No of dependents	*< 2members	24(51.1%)	0.15
	≥ 2members	23(48.9%)	
Level of education	*Upto secondary education (primary and secondary education)	12(25.5%)	< 0.001
	Above secondary education (beyond secondary education)	35(74.5%)	
Family monthly income	*< 50,000 LKR	22 (15.2%)	0.06
	≥ 50, 000 LKR	25 (11.3%)	
Professional engagement	*General public	6(12.8%)	< 0.001
	HCWs	8(17.0%)	
	Military workers	33(70.2%)	

*Reference category (age: <35 years* vs. ≥ 35 years; gender: male* vs. females; number of dependent: ≤ 2 members* vs. ≥ 2 members; level of education: Upto secondary education (primary and secondary education) * vs. above secondary education (beyond secondary education); family monthly income: <50,000 LKR (Sri Lankan Rupees)* vs. ≥ 50,000 LKR; and professional engagement: general public* vs. professionals; HCWs and military professionals)

**p values derived from Chi Square test of independence

HCWs – Healthcare workers

Univariate logistic regression suggested the same variables as significant that were identified with the Chi-square test (Supplementary table).

Multivariate Binary logistic regression revealed that only level of education and professional engagement affected on both depressive symptoms and anxiety (p < 0.01) (Table 5). Having a high level of education was a protective factor for depressive symptoms (Adjusted OR = 0.34) compared to high level of education while being a HCW (Adjusted OR = 4.40) and military personnel ((Adjusted OR = 5.43) were identified as risk factors for depressive symptoms compared to general public. Similarly, having a high level of education was a protective factor for severe anxiety (Adjusted OR = 0.29) compared to high level of education while being a HCW (Adjusted OR = 3.90) and military personnel ((Adjusted OR = 4.52) were identified as risk factors for severe anxiety compared to general public.

**Table 5:** Factors impacted on depressive symptoms and anxiety (n = 367)

Dependent variable	Independent variables	Standard Error	Adjusted OR (95% CI)	*p value
Depressive symptoms	Gender (Females)	0.27	1.02 (0.60–1.72)	0.95
	Level of education (Above Secondary education)	0.28	0.34 (0.20–0.59)	< 0.001
	Professional Engagement - HCWs	0.26	4.40 (2.62–7.38)	< 0.001
	Professional Engagement – Military professionals	0.21	5.43 (2.54–8.47)	< 0.001
Severe Anxiety	Gender (Females)	0.26	1.10 (0.55–1.51)	0.72
	Level of education (Below Secondary education)	0.27	0.29 (0.17–0.49)	< 0.001
	Professional Engagement - HCWs	0.25	3.90 (2.40–6.30)	< 0.001
	Professional Engagement – Military professionals	0.27	4.52 (2.12–7.69)	< 0.001

*p values derived from Multivariate Binary Logistic Regression

Odds Ratio - OR, Confidence Interval – CI, HCWs – Healthcare workers

*Reference category; gender: male* vs. females; level of education: Up to secondary education (primary and secondary education) * vs. above secondary education (beyond secondary education); professional engagement: general public* vs. professionals; HCWs and military professionals

## Discussion

The study observed a greater level of anxiety and depressive symptoms among frontline HCWs and military professionals in Sri Lanka during the pandemic of COVID-19. The general public experienced less anxiety and depressive symptoms when compared to those professionals.

Similar to the finding of HCWs experiencing high levels of depressive symptoms and anxiety, studies done elsewhere also reported consistent findings. A Sri Lankan study conducted with HCWs revealed that 53.3% experienced elevated depressive symptoms while 42.2% experienced mild anxiety, 6.6% with moderate anxiety, and 2.5% with severe anxiety owing to fear of being infectious, spreading to family members, occupational insecurity, and defamation [13]. A review highlighted that during the COVID-19 pandemic, higher level of depression, anxiety, and stress had been reported among non-front line HCWs compared to frontline HCWs, and both groups had higher levels of psychological illness when compared to the general people around the world [4]. De Boni et al. [17] reported that frontline HCWs from Brazil and Spain, highly suffered from depression and anxiety (26.7% of depression, 39.6% of anxiety and 35.4% of both conditions). An Indian study reported a higher prevalence of stress, anxiety, depression, and psychological distress among HCWs compared to the general population [18]. Similarly, a Chinese study reported high level of depressive symptoms among HCWs [19].

HCW’s were the backbone of the COVID-19 prevention and management campaign in Sri Lanka. Their frontline work role could have been contributed for observing more depression and anxiety among them. Apart from that, heavy workload with long duty hours, exhaustion, compulsory quarantine rituals, strict hygienic measures, frequent contact with infected cases, fear of transmission of disease to family members, illness or death of close friends and colleagues, lack of rest and leave and personal isolation associated with COVID-19 may have caused anxiety and depressive symptoms among them. Frontline nurses in several countries, such as India, America, Spain, Australia, and China, also reported of depression due to anxiety and fear of infection, exhaustion because of working for long hours without proper nourishment, lack of medical supplies and resources such as personal protective equipment and lack of communication with patients [20]. A review reported that HCWs verbalized that they had to cope with different psychological challenges, including anxiety, depression and insomnia, due to COVID-19 causing a heavy toll and being affected by different factors, such as exhaustion, personal risk of infection, fear of transmission to family members, illness or death of friends and colleagues, loss of many patients and long shifts combined with unprecedented population restrictions, including personal isolation [21].

In contrast, a study from Singapore showed a considerably low level of anxiety (8.15%) and depression (14.5%) among HCWs [22]. Different contexts of healthcare systems might have different future plans to encounter sudden outbreaks of infectious diseases depending on the infrastructure availability and policy decisions on healthcare provisions. The COVID-19 was the most devastating pandemic experience for Sri Lankans in the 21st century. Therefore, Sri Lankans did not have prepared for it mentally and did not have adequate insight on strict infection control measures in the early stage of the pandemic. In contrast, in Singapore, the healthcare system and people had faced a similar experience during the SARS outbreak. Therefore, their mental preparedness had been already established to tackle the situation and they had followed strong infection control measures even at the early stages of COVID-19 pandemic [22]. Further, previous working experience in infectious diseases of similar nature that have been encountered in the recent past would have been an added advantage [13, 19]. Therefore, HCWs in different countries have a well-planned agenda of facing a pandemic and prior experience to handle it confidently. This may partially reduce the psychological inconvenience they feel in this type of an outbreak.

The depressive symptoms and anxiety among frontline military professionals were also high in this study. Similar to the HCWs, long working hours, compulsory quarantine rituals, strict hygienic measures, separation from family members, and fear of the family members acquiring the disease could have been the possible reasons for high level of depression and anxiety in this group of people. Apart from these, during the pandemic of COVID-19, contribution of military professionals was totally beyond their usual duties and responsibilities. They were assigned to manage the immediate care centers and quarantine centers, screening of infected and suspected cases from the community and mass vaccination programmes. Further the barracks of military professionals created massive clusters of infection that would have increased their psychological disturbance to a greater extent. Unfortunately, we could not find any similar studies done elsewhere focusing on the frontline military personnel in this context.

The general public reported fewer depressive symptoms and anxiety levels compared to professionals in this study. Similarly, De Boni et al., [17] also reported that general household people had low levels of depression and anxiety. A review [18] reported a lesser prevalence of stress, anxiety, depression, and psychological distress in the general population when compared to HCWs in India [18]. The reasons for less prevalence of anxiety and depressive symptoms would have possibly been due to the poor insight regarding the gravity of the COVID 19 pandemic. As reported in a previous study, in Poland, the individuals infected with SARS-like conditions showed a comparably higher rate of persistent thinking and dysfunctional mental health than non-infected persons during the COVID-19 pandemic. When considering Sri Lanka, this is the first-time people explored the pandemic [23]. Therefore, they have poor insight into the gravity of the disease and its consequences. Therefore, people might have lower levels of anxiety and depression. Studies elsewhere found worsening of a pre-existing psychiatric condition, female gender, occupation or professional engagement, previous exposure to trauma, working remotely and age are associated with higher risks for psychological disturbance, post-traumatic stress disorders (PTSD), and depression due to COVID-19 among the general public [24, 25]. However, we were unable to see any associations except in professional engagement and level of education (low level of education). Different factors revealed in different studies might be related to the sample size and social factors such as prior experience with the pandemics, awareness, psychological status, and support.

This study found significant information about the psychological disturbances among the frontline healthcare and military workforce in Sri Lanka. Mental well-being among this frontline health and security workforce is a determinant factor in the management of many crucial issues in Sri Lanka in the future, similar to the COVID-19 pandemic. Therefore, these findings are important to make a few critical decisions on their health, including all the aspects such as physical, mental, social, and environmental. A long-term plan to resolve such issues and to eliminate this important workforce becoming psychologically ill should be done using these findings as a foundation. Having appropriate mental health programmes, strict epidemic prevention and control policy, community mental health service system, online mental health service, telemedicine, and medical services for them would be a few measures that can be taken to resolve the issue. Due to the non-availability of research findings on military personnel, these findings would be more important to be aware of the psychological disturbances among military personnel who worked at the frontline during the COVID-19 period.

However, this study has a few limitations including, the non-probability sampling approach and fairly a lesser number of participants. The movement restrictions enforced during the study period did not allow a valid method of sampling with more participants. Therefore, this limitation is seen in many other studies done during this period. Further, we did not fit the models for each group since the sub-group analysis did not reveal the significant associations, possibly due to the limited participants in each sub-group. Also, we collected a limited number of essential information during the data collection considering the feasibility during the pandemic. However, the actual psychological burden might influence extraneous variables that has not been addressed in our study. These may limit the generalizability of findings. Therefore, it would be better if a future study considers a more representative sample covering all the areas of Sri Lanka with a reasonably higher number of samples.

## Conclusions

The current study revealed a greater level of anxiety and depressive symptoms among frontline HCWs and military professionals in Sri Lanka during the pandemic of COVID-19 compared to the general public. The professional engagement (being a HCW or military professional) was the major risk factor identified that impacted the level of depressive symptoms and anxiety. Having a high level of education was seen as a protective factor for getting depressive symptoms and severe anxiety. These findings would lay the foundation for identifying risk groups for psychological disturbance and psychological first aid for risk groups to better deal with mental problems. An emergency preparedness plan to deal with sudden outbreaks of infectious situation is also important.

## Electronic supplementary material

Below is the link to the electronic supplementary material.

Suppleantery Material 1 Univariate analysis of risk factors of depressive symptoms and severe anxiety of participants (n=367)

## Data Availability

The data used to support the findings of this study are available from the corresponding author upon request.

## Abbreviations

COVID-19: Coronavirus disease
HCWs: Healthcare workers
PDS: Peradeniya depression scale
GAD: Generalized Anxiety Disorder
PTSD: Post traumatic stress disorders
OR: Odds Ratio
CI-95%: Confidence interval
SD: Standard deviation
LKR: Sri Lankan Rupees
