# Supplementary material for: Psychological disturbances encountered by the healthcare professionals, military professionals and general public in Sri Lanka during COVID-19 pandemic: a cross-sectional study
Source: BMC Psychiatry. 2023 Jun 21;23:452. doi: 10.1186/s12888-023-04918-2 (PMC10283207; doi:10.1186/s12888-023-04918-2)
Supplement: Supplementary file 1 — Suppleantery Material 1 Univariate analysis of risk factors of depressive symptoms and severe anxiety of participants (n=367) [file 12888_2023_4918_MOESM1_ESM.docx]

Supplementary Table – Univariate analysis of risk factors of depressive symptoms and severe anxiety of participants (n=367)

| Factor | Standard error | P value | Odds Ratio (OR) | 95% CI of OR | |
| --- | --- | --- | --- | --- | --- |
|  |  |  |  | Lower | Upper |
| *Depressive symptoms* |  | | | | |
| Gender (female) | .216 | .014 | 1.588 | .385 | 2.897 |
| No. of dependents (≥2 members) | .214 | .040 | 1.948 | .624 | 2.442 |
| Level of education (Above secondary education (beyond secondary education) | .218 | .000 | .448 | .292 | .687 |
| Being a HCW | .305 | .000 | 1.101 | .056 | 2.184 |
| Being a Military professional | .295 | .000 | 1.158 | .088 | 2.281 |
| *Severe anxiety* |  | | | | |
| Gender (female) | .212 | .034 | 1.639 | .422 | .967 |
| Level of education (Above secondary education (beyond secondary education) | .216 | .000 | .402 | .263 | .615 |
| Being a HCW | .364 | .000 | 1.076 | .037 | 2.156 |
| Being a Military professional | .365 | .000 | 1.103 | .051 | 2.211 |
| HCW = healthcare worker  *Reference category; gender: male* *vs* females; number of dependent: < 2 members* vs ≥ 2 members; level of education: Upto secondary education (primary and secondary education) * *vs* above secondary education (beyond secondary education); and professional engagement: general public* *vs* professionals; HCWs and military professionals | | | | | |
